# A Dominant-Negative Approach That Prevents Diphthamide Formation Confers Resistance to *Pseudomonas* Exotoxin A and Diphtheria Toxin

**DOI:** 10.1371/journal.pone.0015753

**Published:** 2010-12-23

**Authors:** Vincent Roy, Karim Ghani, Manuel Caruso

**Affiliations:** Le Centre de Recherche en Cancérologie de l'Université Laval, L'Hôtel Dieu de Québec, Centre Hospitalier Universitaire de Québec, Québec, Canada; Charité-University Medicine Berlin, Germany

## Abstract

Diphtheria toxin (DT), *Pseudomonas aeruginosa* Exotoxin A (ETA) and cholix toxin from *Vibrio cholerae* share the same mechanism of toxicity; these enzymes ADP-rybosylate elongation factor-2 (EF-2) on a modified histidine residue called diphthamide, leading to a block in protein synthesis. Mutant Chinese hamster ovary cells that are defective in the formation of diphthamide have no distinct phenotype except their resistance to DT and ETA. These observations led us to predict that a strategy that prevents the formation of diphthamide to confer DT and ETA resistance is likely to be safe. It is well documented that Dph1 and Dph2 are involved in the first biochemical step of diphthamide formation and that these two proteins interact with each other. We hypothesized that we could block diphthamide formation with a dominant negative mutant of either Dph1 or Dph2. We report in this study the first cellular-targeted strategy that protects against DT and ETA toxicity. We have generated Dph2(C-), a dominant-negative mutant of Dph2, that could block very efficiently the formation of diphthamide. Cells expressing Dph2(C-) were 1000-fold more resistant to DT than parental cells, and a similar protection against *Pseudomonas* exotoxin A was also obtained. The targeting of a cellular component with this approach should have a reduced risk of generating resistance as it is commonly seen with antibiotic treatments.

## Introduction

Diphtheria toxin (DT) and *Pseudomonas aeruginosa* exotoxin A (ETA) are two bacterial A-B toxins that share the same mechanism of toxicity. These toxins are characterized by a B moiety that recognizes the cell surface receptor but that also plays a role in the translocation of the toxin into the cytosol, and an A moiety that contains the catalytic activity of the toxin. After binding to its receptor and endocytosis, the A subunit of DT enters the cytosol from the acidic endosomes and the one from ETA is released from the endoplasmic reticulum. The A subunit can then inactivate the elongation factor-2 (EF-2) by adenosine diphosphate (ADP)-ribosylating a modified histidine residue called diphthamide leading to cell death by blocking protein translation [1], [2]. More recently, it was reported that cholix toxin from *Vibrio cholerae* has also a similar ADP-ribosylating activity on EF-2 [3].

*P. aeruginosa* is a Gram-negative bacillus ubiquitously present in the environment and, according to the Central for Disease Control, it is the fourth most commonly isolated nosocomial pathogen [4], [5]. Nearly all *P. aeruginosa* clinical cases can be associated with compromised host defence. Systemic infections are common in patients with severe burns, and immunosuppressed AIDS and cancer patients. The infection by *P. aeruginosa* can also be seen with contact lenses wearers that develop keratitis of the cornea. *P. aeruginosa* is also responsible for ventilator-acquired peneumonia, and it is the primarily cause of mortality in cystic fibrosis patients due to lung infection [4]. The pathogenicity of *P. aeruginosa* is associated to several virulence factors but ETA is produced by 95% of *P. aeruginosa* clinical isolates and it is the most toxic [6]. It has been reported that ETA deficient strains are less pathogenic in mice than wild-type strains [7], [8], and that the immunization directed against ETA increased survival in normal and thermally injured mice infected by *P. aeruginosa* [9]–[14]. It is also clear that ETA contributes to the organism's pathogenicity in keratitis [15], [16]. Overall, the high incidence of this microorganism, the severity of its infection and the resistance to antimicrobial treatments promote *P. aeruginosa* as a major human pathogen.

The implementation of a vaccine program in the 1940s and 1950s based on diphtheria toxoid had nearly eliminated diphtheria in developing countries. However, recent outbreaks of diphtheria have been reported in countries like Russia, in newly independent states of the former Soviet Union, and in poor socio-economically disadvantaged groups living in crowded conditions in Europe and the US [17], [18]. One reason for the re-emergence of epidemics in countries where immunization have been performed has been explained by a lack of exposure to toxigenic strains of diphtheria necessary for the boost and maintenance of immunity against this pathogen [17]. It is well documented that the level of immunity declines in late childhood and adolescence, and some serological surveys demonstrated that more than 50% of adults lack immunity to DT in some industrialized countries [17], [19], [20]. The introduction of a new biotype of toxigenic *C. diphtheriae* could also be a factor in the re-emergence of diphtheria [17], [21]. It is now admitted that a lack of immunity against diphtheria in adults represents a potential threat that could lead to the development of epidemics in industrialized countries [17], [21].

The molecular target of DT, ETA and cholix toxin is the diphthamide residue whose biosynthesis consists of stepwise modifications of a histidine residue present at position 715 (699 in yeast) [22]–[27]. Five proteins named Dph1 to Dph5 are involved in this process in yeast and eukaryotic cells [28]–[31]. The chemical transformation into diphthamide requires three successive biochemical reactions starting with the transfer of a 3-amino-3-carboxypropyl from AdoMet to the imidazole C-2 of the histidine residue [25]. Studies in yeast and Chinese hamster ovary (CHO) cells indicated that Dph1 to Dph4 are implicated in the first biochemical transformation [29]–[31]. More recently, WDR85 was identified as the fifth protein involved in the first step of diphthamide formation [32]. In the second step, Dph5 is involved in the trymethylation of the diphthamide intermediate [28] and, up to now, no protein involved in the last step of the diphthamide biosynthesis has been identified. Diphthamide can be found in all eukaryotic organisms and in archaebacteria except eubacteria, suggesting a relevant role in cell physiology [33], [34]. The diphthamide residue is located at the tip of a domain loop in EF2 that mimics the anticodon loop of a tRNA. It has been suggested that diphthamide stabilizes the tRNA-anticodon-mRNA-codon interaction and is necessary to maintain the translation reading frame [35], [36]. Nevertheless, except for yeast *Dph3* mutants, the other yeast and CHO cell lines deficient in some of the *Dph* genes do not show distinctive phenotypes except for DT and ETA resistance [27], [31], [37]. We thus reasoned that the blockade of diphthamide formation could be an efficient therapeutic strategy for treating *P. aeruginosa* or *C. diphtheriae* infections.

Dph1 and Dph2 interact *in vivo* in yeast as well as in eukaryotic cells, and it has also been suggested that these two proteins could be part of a catalytic complex involved in the first step of diphthamide synthesis [31], [38]. We then hypothesized that it could be possible to generate a mutant of Dph1 or Dph2 that could inhibit in a dominant manner the formation of diphthamide. We report in this study the identification of a C-terminal deletion mutant of Dph2 that could completely protect cells against the toxicity of DT and ETA.

## Results

### A C-terminal Dph2 Deletion Mutant Protects Cells Against DT Toxicity

**Figure 1:**
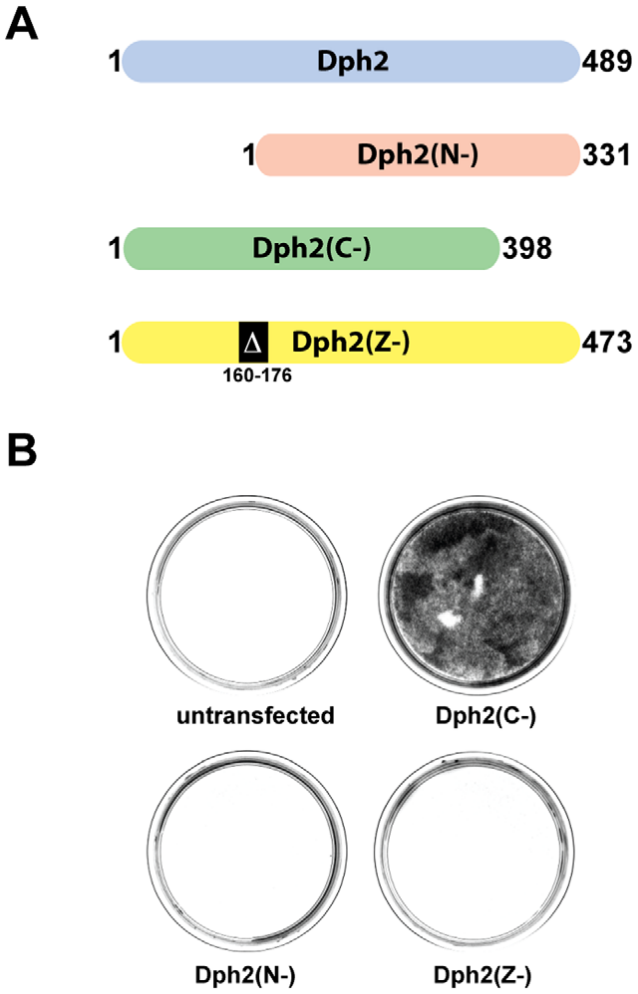
A C-terminal Dph2 deletion mutant protects against DT toxicity. A, Schematic drawings of Dph2 and Dph2 deletion mutants. B, Dph2(C-) confers DT resistance in TE671 cells. TE671 cells untransfected and transfected with plasmids expressing *Dph2(C-)*, *Dph2(N-)* and *Dph2(Z-)* were selected with DT for 7 days. Colonies were then fixed with methanol and stained with methylene blue. The colony assay was performed three times; one representative experiment is displayed.

Deletion mutants of Dph2 were generated in order to create a dominant negative protein that could block the first step of diphthamide formation. First, the hamster *Dph2* cDNA gene was cloned by PCR amplification using degenerated primers based on the mouse and the human sequence. A strong homology was found with the mouse and human *Dph2* nucleotide sequence with 89% and 82% identity, respectively. Three deletion mutants were constructed: a 158 amino acid N-terminal deletion mutant; Dph2(N-), a 91 amino acid C-terminal deletion mutant; Dph2(C-), and a 16 amino acid deletion mutant between residues 160–176; Dph2(Z-) (Fig. 1A). The Dph2(Z-) mutant was designed using the “PROSITE” program that identified a putative leucine zipper motif between residues 160–181 [39]. These mutants were then transfected into TE671 cells followed by DT selection. All the cells transfected with the N- and the Z-deletion mutants were killed by DT, like the untransfected control cells. On the contrary, the plate transfected with *Dph2(C-)* gave rise to a high number of DT-resistant colonies, indicating that this mutant was able to protect cells against DT toxicity (Fig. 1B). Similar results were also obtained with other human cell lines and CHO cells (data not shown). We are also in the process of defining the shortest deletion mutant that could confer resistance to DT and ETA: preliminary results indicate that the same human Dph2 deletion mutant is functional but not a shorter version with a 133 amino acid deletion (data not shown).

### Cells Expressing Dph2(C-) and Cells Dph2-deficient are Equally Resistant to DT

**Figure 2:**
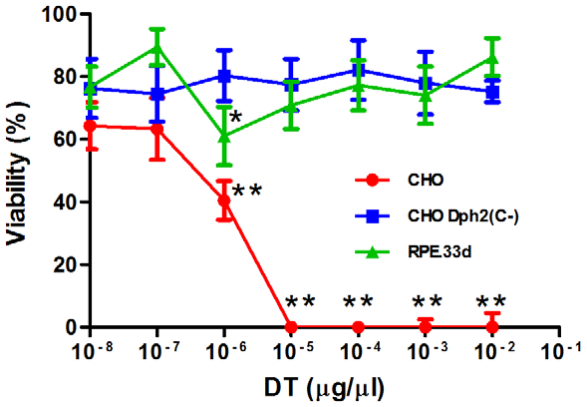
CHO cells expressing Dph2(C-) are highly resistant to DT. DT dose response was measured in a cell proliferation assay. Data are expressed as a proliferation percentage relative to the proliferation of cells in the absence of drug. Data are the average of at least four values ± s.d. from one representative experiment that was reproduced twice. Statistical significance was assessed by one-way ANOVA followed by Dunnett's multiple comparison posttest. The values of each point in the curves are compared to those obtained with the 10^−8^ µg/µl DT concentration (**, *p*<0.01; *, *p*<0.05).

In order to investigate to which extent Dph2(C-) could protect cells against DT, CHO cells expressing Dph2(C-) were cultured with increasing concentrations of DT up to 10^−2^ µg/µl. There was no significant effect on the proliferation of cells expressing Dph2(C-) even if they were cultured with the highest dose of DT. These cells were as resistant to DT as RPE.33d that is a diphthamide-negative CHO subline defective for Dph2 [27]. Wild-type CHO cells were killed with 10^−5^ µg/µl of DT (Fig. 2). These data demonstrated a strong potency of Dph2(C-) in protecting cells against DT toxicity.

### Dph2(C-) Confers Resistance to ETA

**Figure 3:**
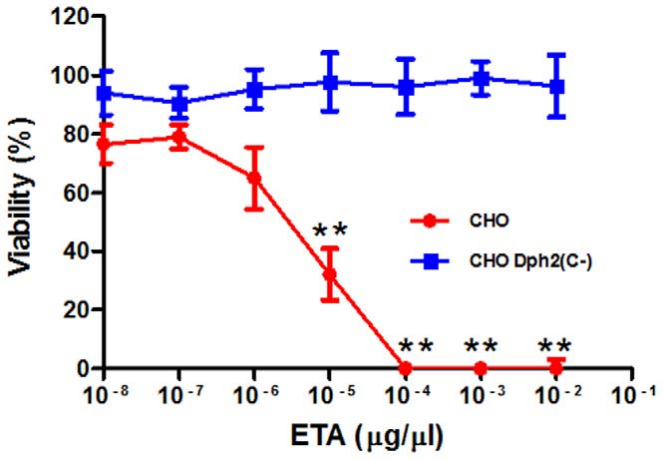
CHO cells expressing Dph2(C-) are resistant to ETA. ETA dose response was measured in a cell proliferation assay. Data are expressed as a proliferation percentage relative to the proliferation of cells in the absence of drug. Data are the average of at least four values ± s.d. from one representative experiment that was reproduced twice. Statistical significance was assessed by one-way ANOVA followed by Dunnett's multiple comparison posttest. The values of each point in the curves are compared to those obtained with the 10^−8^ µg/µl ETA concentration (**, *p*<0.01).

We next tested the ability of Dph2(C-) to protect against ETA. Proliferation experiments with increasing ETA concentrations were performed on CHO cells that had been transfected with *dph2(C-)* and selected with DT. These cells were completely resistant to ETA at concentrations as high as 10^−2^ µg/µl, while the viability of parental CHO cells was reduced by 70% with as little as 10^−5^ µg/µl of the toxin (Fig. 3). This result indicated that DT protection conferred by Dph2(C-) could be extended to ETA.

### Dph2(C-) Prevents the ADP-ribosylation of EF-2 by DT

**Figure 4:**
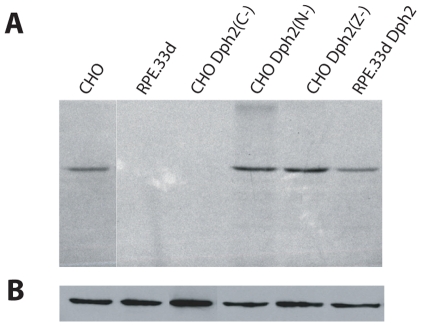
Absence of ADP-rybosylation by DT in CHO cells expressing Dph2(C-). A, Autoradiography of cellular extracts that underwent an ADP-rybosylation assay. B, Western blot with an antibody directed against EF-2. The lane between the CHO lane and the RPE.33d lane was not relevant and has been deleted. One representative experiment out of two is displayed.

The mechanism of toxin protection conferred by Dph2(C-) was investigated *in vitro* using an EF-2 ADP-ribosylation assay. Protein extracts from different CHO cell lines were incubated with DT and radiolabeled nicotinamide adenine dinucleotide (NAD), and run in an SDS-PAGE gel. As shown by autoradiography, EF-2 was ADP-ribosylated in parental CHO cells and in CHO cells transfected with the inactive N- and Z-deletion mutants. However, no EF-2 ADP-ribosylated form could be detected in CHO cells expressing Dph2(C-), indicating that the absence of diphthamide was responsible for the lack of DT toxicity. As expected, EF-2 from RPE.33d cells could not be ADP-ribosylated but the enzymatic activity of DT could be detected after transfection of *dph2* in those cells (Fig. 4A). EF-2 was present in similar amounts in all tested samples as shown by Western blot (Fig. 4B).

### Dph2(C-) Binds Dph1 and Competes with Wild-type Dph2

**Figure 5:**
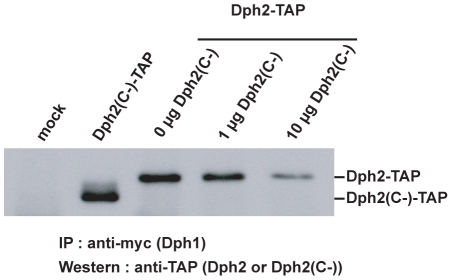
Dph2(C-) binds to Dph1 and competes with wild-type Dph2. Western blot analysis performed with an anti-TAP antibody of cellular extracts immunoprecipitated with an anti-myc antibody. Lane 1, untransfected 293T cells were used as control; lane 2, pcDNA-Dph1-myc and pNC-Dph2(C-)-TAP plasmids were cotransfected; lane 3–5, pcDNA-Dph1-myc and pNC-Dph2-TAP plasmids were cotransfected with 0, 1, and 10 µg of Dph2(C-) plasmid. One representative experiment out of two is displayed.

One possible explanation for the Dph2(C-) anti-toxin activity was a competition with endogenous Dph2 for the binding to Dph1, leading to the block of the first biochemical step of diphthamide formation. Cotransfection/immunoprecipitation experiments showed that Dph2(C-) fused to a TAP-tag could be immunoprecipitated by an anti-myc antibody recognizing a Dph1-myc fusion protein (Fig. 5; lane 2). Furthermore, cotransfection of Dph2-TAP and Dph2(C-) expression plasmids in a 1∶1 ratio (Fig. 5; lane 4) decreased the amount of Dph2-TAP recovered by immunoprecipitation with the anti-myc antibody (Fig. 5; lane 4 versus lane 3). With a 10-fold excess of the Dph2(C-) plasmid over the Dph2 plasmid, the band corresponding to Dph2-TAP on the Western blot was barely detected (Fig. 5; lane 5). These results clearly showed that Dph2(C-) binds to Dph1, and that it competes with wild-type Dph2.

## Discussion

In addition of being the target of DT and ETA, it is assumed that the diphthamide residue present on EF-2 plays an important biological role since it is present in all eukaryotic organisms and in archaebacteria [33], [34]. Nevertheless, CHO cells mutated for diphthamide formation are able to synthesize proteins and grow as well as wild-type cells [27], [31]. These observations led us to predict that a strategy that prevents the formation of diphthamide to confer DT and ETA resistance is likely to be safe. In the present study, we have designed a dominant-negative strategy that could prevent diphthamide formation and that could render cells resistant to DT and ETA toxicity. We found that Dph2(C-), a C-terminal deletion mutant of Dph2, could completely block DT and ETA induced toxicity by preventing the ADP-rybosylation of EF-2. Dph2(C-) was able to bind Dph1 and to compete with wild-type Dph2, suggesting that the first step of diphthamide formation was impaired.

All Dph proteins involved in the biosynthesis of diphthamide have been well studied. *Dph1* is a candidate tumor suppressor gene that has been cloned independently as *Ovca1*; a loss of *Dph1* heterozygosity is frequent in breast and ovarian carcinoma and is associated to a decrease in protein expression [40]. *Ovca1* heterozygote mice develop spontaneous cancer, thus, confirming the suppressor function of Dph1. Dph1 is also essential to mouse development as the *Ovca1^−/−^* mice die during embryonic development and at birth with developmental delay and defects in multiple organ systems. Moreover, embryonic fibroblasts from these mice revealed a role of Dph1 in the regulation of cell proliferation [40]. The phenotype of Dph4-null mutants is similar to *Ovca1^−/−^* mice but heterozygous mice for the Dph4 mutation do not develop tumors, suggesting an additional tumor suppressive function for Dph1 [41]. In addition to DT resistance, yeast *Dph3* mutants show defect in growth and drug, and temperature sensitivity suggesting a broader biological role for Dph3 [31], [42]. *Dph3* knockout mice have also been generated and are associated with lethality during embryonic life [43]. Dph3 has a role outside diphthamide formation that could account for the severe phenotypes observed in knockout mice [44]. It is also most likely that a *Dph2* null mutation in mice would be lethal before or at birth. The generation of a transgenic mouse with a conditional expression of Dph2(C-) could circumvent the possible lethality associated to the absence of the wild-type gene and allow the study of the role of the diphthamide residue in adult tissue. This mouse model could be used to assess the efficacy of Dph2(C-) in the treatment of *P. aeruginosa* infections.

From a therapeutic stand-point, it would be feasible to deliver the *Dph2(C-)* gene directly at sites of *P. aeruginosa* infection. For example, viral or non viral delivery systems could be used to introduce *Dph2(C-)* in corneal cells as a local treatment for refractory *P. aeruginosa*-induced keratitis [45]. A similar gene therapy strategy could also be offered via aerosol to cystic fibrosis patients that develop *P. aeruginosa* infections in the lungs [46] or for topical treatments of wounds in burn patients [47]. The crystal structure of a Dph1/Dph2(C-) complex may help in the development of small molecules with similar therapeutic activity as Dph2(C-). High-throughput screening technologies with chemical libraries could also lead to drug candidates for the treatment of *P. aeruginosa* and *C. diphtheria* infection.

Dph2(C-) might also be useful for the treatment of other pathogenic bacteria. Indeed, cholix toxin from *Vibrio cholerae* has the same mechanism of toxicity of DT and ETA [3], and putative ADP-rybosyltransferases with a diphthamide residue as target have also been identified in *Neisseria gonorrhoeae* and *Staphilococcus aureus* [48]. Finally, efforts are being made to develop antidotes to counter the health consequences of bioterrorism; dominant-negative mutants of anthrax toxin have already been identified [49], and Dph2(C-) could also be a valuable asset against potential ETA- or DT-based bioweapons.

In conclusion, our results indicate that it is possible to completely abolish DT and ETA toxicity by blocking the formation of diphthamide with a dominant-negative strategy. This is the first demonstration that the targeting of a cellular component can protect against DT and ETA toxicity. Dph2(C-) could have a broad range of clinical applications, including its use alone or in combination with standard antibiotics for the treatment of *P. aeruginosa* and *C. diphtheriae* infection. This approach is likely to have a reduced risk of generating resistance, a common outcome with antibiotic therapy.

## Materials and Methods

### DNA Constructions

The hamster *Dph2* gene was amplified by RT-PCR from CHO-K1 cells using the mouse 5′ primer **Dph2E-5′** 5′-ATCGAATTCATGGAGTCTACGTTCAGCAG-3′ containing a EcoRI site (underlined) and a 3′ degenerated primer designed according to the mouse and human *Dph2* sequence 5′-TCAGCNGCTNCCCTCATC-3′. The pBS-Dph2 plasmid was then obtained by cloning the PCR product in pBluescript SK+ (Stratagene, La Jolla, CA) opened in EcoRI/EcoRV. The hamster *Dph2* gene was sequenced and deposited to GenBank (accession no. DQ981502). Three deletion mutants of *Dph2* were constructed by PCR using pBS-Dph2 as template, and they were cloned in the eukaryotic vectors pMD2iPuro^r^, pcDNA3-TAP and pNC described elsewhere [50]–[52].

For *Dph2*, a PCR was performed with the following primers: **Dph2K-5**′ 5′-CGGGGTACCATGGAGTCTACGTTCAG-3′ containing a KpnI site (underlined) and a **Dph2B-3**′ 3′ primer 5′-CGCGGATCCGCCGCTGCCCTCATCCT-3′ containing a BamHI site (underlined). The KpnI/BamHI digested PCR product was then cloned in pcDNA3-TAP opened in KpnI/BamHI to create the pcDNA-Dph2-TAP plasmid.

The C-terminal mutant was constructed by PCR with the 5′ primer **Dph2K-5**′ and the 3′ primer 5′-GAATTCGGGAGTGGAACATA-3′. The pcDNA-Dph2(C-)-TAP plasmid was obtained by cloning the KpnI digested PCR fragment in pcDNA3-TAP opened in BamHI blunted by klenow and KpnI.

pNC-Dph2-TAP and pNC-Dph2(C-)-TAP were generated by cloning in pNC opened in BamHI Dph2-TAP and Dph2(C-)-TAP linked to a BamHI adaptor.

The pMD2-Dph2iPuro^r^ was constructed by cloning in pMD2iPuro^r^ opened in EcoRI/XhoI Dph2 from pBS-Dph2 digested by EcoRI/XhoI.

The pMD2-Dph2(C-)iPuro^r^ plasmid was constructed by inserting a Dph2(C-) PCR product with the 5′ primer **Dph2E-5**′ and the 3′ primer 5′-TTAATTCGGGAGTGGAACAT-3′ digested by EcoRI and inserted in pMD2iPuro^r^ opened by EcoRI/EcoRV.

For the construction of the N-terminal mutant, the PCR amplification was performed using the 5′ primer 5′-CGCGGTACCATGGAGCCAGCTTGTGC-3′ containing a KpnI site (underlined) and the 3′ primer **Dph2B-3**′. The PCR product was digested by KpnI and BamHI and ligated in the pcDNA3-TAP plasmid to give the pcDNA-Dph2(N-)-TAP plasmid.

The leucine zipper mutant (Z-) was created using two different PCR fragments ligated together in pcDNA3-TAP. The first segment was amplified with the 5′ primer **Dph2K-5**′ and the 3′ primer 5′-GGCATGGGCACAAGCTG-3′, and the second fragment was amplified with the 5′ primer 5′-ATCTCCAGCCCAGCTCTT-3′ and the 3′ primer **Dph2B-3**′. The pcDNA-Dph2(Z-)TAP plasmid was constructed by cloning the first amplification product digested by KpnI and the second one digested by BamHI in pcDNA3-TAP.

The pMD2-Dph2(N-)iPuro^r^ and pMD2-Dph2(Z-)iPuro^r^ plasmids were constructed similarly. The KpnI/BamHI fragment from pcDNA-Dph2(N-)-TAP or pcDNA-Dph2(Z-)-TAP was blunted klenow and ligated in pMD2iPuro^r^ vector digested by EcoRV and XbaI blunted by klenow. A stop codon was created at the C-terminal end due to the cloning procedure.

The mouse *Dph1* gene was amplified by RT-PCR using cDNAs prepared from mouse PG13 cells with the following primers: the 5′ primer 5′-ATGGCGGCGCTGGTA-3′ and the 3′ primer 5′-CGCGGATCCGGGAGCCGGCGAAGTA-3′ containing a BamHI site (underlined). The PCR product was then digested by BamHI and ligated in pcDNA3.1(-)/myc-His A (Invitrogen, Carlsbad, CA) to create the pcDNA-Dph1-myc vector.

### Tissue Culture and Transfections

CHO K1 cells (ATCC CCL-61) were grown in RPMI medium (Invitrogen, Carlsbad, CA), and TE671 (ATCC CRL-8805), RPE.33d [27], HeLa (ATCC CCL-2) and 293T [53] cells were cultured in Dulbecco's modified Eagle's medium (Sigma, Oakville, Canada). Both media were supplemented with 10% fetal calf serum (PAA laboratories, Etobicoke, Canada) and antibiotics.

Transfections of CHO K1 and HeLa cells were performed either by the calcium phosphate method or using linear Polyethylenimine (PEI; Polysciences, Warrington, PA), whereas the RPE.33d cells were only transfected with PEI. For the PEI transfection, 2 µg of PEI per µg of DNA are mixed in 500 µl of serum free DMEM for 15 min. The mixture is then added to subconfluent cells in a 60-mm culture dish containing 5 ml of DMEM with 10% fetal calf serum. The transfection mixture is kept overnight until the following day. For the generation of stable transfectants, CHO K1 and RPE.33d were selected with puromycin at 10 µg/ml and 5 µg/ml, respectively, and a concentration of 5×10^−5^ mg/ml was used for the selection of cells with DT (List Biological laboratories, Campbell, CA).

### Cell Proliferation Assay [54]

The cells were plated at a concentration of 3×10^3^ cells/well in 96-well plates in six replicates for each toxin concentration. The next day, increasing concentrations of DT or ETA (List Biological laboratories) were added to the wells for 3 days. Cell proliferation was then measured by the 3-(4,5-dimethylthiazol-2-yl)-2,5-diphenyltetrazolium bromide (MTT) (Sigma) assay, which consists of adding 37.5 µl of MTT (1 mg/ml) to the 150 µl of medium in each well for 4 hours at 37°C. After gently removal of the medium, 150 µl of dimethyl sulfoxide (DMSO) was added and plates were gently shaken for 10 minutes to dissolve the formazan blue crystals. The absorbance was then measured at 595 nm with a microplate reader (Tecan, Research Triangle Park, NC).

### ADP-ribosylation Assay

Confluent cells grown in 60-mm plates were lysed in 400 µl of modified RIPA buffer (50 mM Tris-HCl, pH 7.4, 1% Nonidet P-40, 0.25% sodium deoxycholate, 150 mM NaCl, 1 mM EDTA and protease inhibitors) at 4°C for 45 minutes. Cell extracts were then centrifuged at 13,000×g for 30 minutes at 4°C, and the protein concentration of the supernatant was determined by the Bradford protein assay (Biorad, Hercules, CA). DT was nicked by mixing together 27 µl of DT (1 mg/ml) with 3 µl trypsin (10 mg/ml) for 15 minutes. The reaction was stopped by adding 1 µl of protein inhibitors (Sigma, P8340). For the ADP-ribosylation assay, 100 µg of protein extract were mixed with 500 ng nicked DT, 2 µl [C^14^]NAD, 288 mCi/mmol (GE Healthcare, Baie d'Urfe, Canada), 20 mM Tris-HCL, pH 7.5, 50 mM dithiothreitol (DTT), 1 mM EDTA at 30°C for 30 minutes in a final volume of 120 µl. From the 120 µl reaction, 50 µl were mixed with 17 µl of 4X sample buffer (200 mM Tris-HCL, 8% SDS, 0.4% bromophenol blue, 40% glycerol, 400 mM DTT) and analysed by SDS-PAGE on a 10% acrylamide gel followed by autoradiography.

### Immunoprecipitation

293T cells plated in 60-mm plates were transfected with 1 µg of the pcDNA-Dph1-myc plasmid with 1 µg of pNC-Dph2-TAP, and with 0, 1 or 10 µg of pMD2-Dph2(C-) plasmids. One µg of the pcDNA-Dph1-myc plasmid with 1 µg of pNC-Dph2(C-)-TAP were also transfected to assess the binding of Dph2(C-) with Dph1. Two days post-transfection, cells were lysed in 500 µl of E1A buffer (20 mM HEPES pH7.9, 250 mM NaCl, 0.1% IGEPAL, 10% glycerol, 1 mM β-mercaptoethanol and protease inhibitors) at 4°C for 30 minutes. The lysis product was then incubated with 1 µg of the α-myc antibody (clone 9E10) (Sigma) at 4°C for 4 hours. Following this incubation, the extracts were treated with protein A-Sepharose (GE Healthcare) at 4°C for 1 hour. The beads were then washed three times with E1A buffer and ressuspended in 40 µl loading buffer (50 mM Tris-Cl pH6.8, 2% SDS, 0,1% bromophenol blue, 10% glycerol, 100 mM DTT). The samples were then analyzed by Western blot.

### Western Blot Analysis

Cell extracts prepared for the ADP-ribosylation assays were also analysed by Western blot. Thirty µg of total protein extract were mixed with sample buffer (as described above) and loaded on a 10% SDS-polyacrylamide gel and separated by electrophoresis. The proteins were transferred on nitrocellulose membranes (GE Healthcare) followed by Western blotting using a goat antibody directed against a linear peptide of the carboxyl terminus of EF-2 (Santa Cruz Biotechnology, Santa Cruz, CA). For the immunoprecipitation experiment, the samples were analysed with an anti-TAP antibody (Open Biosystems, Huntsville, AL). The reactive bands were detected using the Western Lightning Chemiluminescence Reagent Plus kit (Perkin Elmer Life Sciences, Boston, MA).

### Statistical Analysis

One-way ANOVA followed by Dunnett's multiple comparison posttest was performed with GraphPad Prism (GraphPad Software Inc., San Diego, CA) on data presented in figures 2 and 3.
